# INCREASING DEPRESSION PREVALENCE AMONG OLD ADULTS DURING THE COVID-19 PANDEMIC

**DOI:** 10.1093/geroni/igad104.3430

**Published:** 2023-12-21

**Authors:** Jiangyuan Zhu, Guohua Li, Barbara Lang, Stanford Chihuri

**Affiliations:** Columbia University Medical Center, New York City, New York, United States; Columbia University, New York City, New York, United States; Columbia University, New York City, New York, United States; Columbia University, New York City, New York, United States

## Abstract

Previous research has reported worsening mental health outcomes in children and young adults amid the COVID-19 pandemic. The purpose of this study was to assess the impact of the COVID-19 pandemic on the prevalence of depression in older adults. The Longitudinal Research on Aging Drivers (LongRoad) study involved prospectively following (between July 2015 and September 2022) 2990 drivers aged 65-79 years recruited from five sites across the contiguous United States. Study participants were assessed at baseline and annually during the follow-up period using the Patient-Reported Outcomes Measurement Information System (PROMIS). The overall response rate was 69.1%. The prevalence of depression increased from 7.12% during 2015-2019 to 9.89% during 2020-2022 (p < 0.001). After adjusting for age, gender, race and ethnicity, marital status, and annual household income, the risk of depression increased by 42% [adjusted odd ratio (aOR) 1.422, 95% confidence interval 1.237-1.635] during the COVID-19 pandemic. The results of this study indicate that the prevalence of depression among older adults increased significantly during the COVID-19 pandemic.

